# Undergraduate medical education in general practice/family medicine throughout Europe – a descriptive study

**DOI:** 10.1186/1472-6920-13-157

**Published:** 2013-12-01

**Authors:** Mette Brekke, Francesco Carelli, Natalia Zarbailov, Givi Javashvili, Stefan Wilm, Markku Timonen, Howard Tandeter

**Affiliations:** 1 Department of General Practice, Institute of Health and Society, University of Oslo, P.O. Box 1130, Blindern, 0318 Oslo, Norway; 2 University of Milan, Milan, Italy; 3 University Campus BioMedico in Rome, Rome, Italy; 4 Department of Family Medicine, State Medical and Pharmaceutical University “Nicolae Testemitanu”, 165 bd. Stefan Cel Mare si Sfint, 2004 Chisinau, Moldova; 5 Department of Family Medicine, Tbilisi State Medical University, 33 Vazha-Phshavela Avenue, Tbilisi 0177 Georgia; 6 Institute of General Practice, Medical Faculty, Heinrich Heine University, Moorenstr. 5, 40225 Duesseldorf, Germany; 7 Institute of Health Sciences, University of Oulu, P.O. Box 5000, FIN-90014 Oulu, Finland; 8 Department of Family Medicine, Ben Gurion University, P.O. Box 653, 84105 Beer Sheva, Israel

**Keywords:** General practice/family medicine, Medical education, Undergraduate, Clinical curriculum, Europe

## Abstract

**Background:**

It is increasingly becoming evident that a strong primary health care system is more likely to provide better population health, more equity in health throughout the population, and better use of economic resources, compared to systems that are oriented towards specialty care. Developing and maintaining a strong and sustainable primary health care requires that a substantial part of graduating doctors go into primary care. This in turn requires that general practice/family medicine (GP/FM) strongly influences the curricula in medical schools. In the present paper we aim at describing the extent of GP/FM teaching in medical schools throughout Europe, checking for the presence of GP/FM curricula and clinical teaching in GP offices.

**Methods:**

A brief questionnaire was e-mailed to GP/FM or other professors at European medical universities.

**Results:**

259 out of 400 existing universities in 39 European countries responded to our questionnaire. Out of these, 35 (13.5%) reported to have no GP/FM curriculum. These 35 medical faculties were located in 12 different European countries. In addition, 15 of the medical schools where a GP/FM curriculum did exist, reported that this curriculum did not include any clinical component (n = 5), or that the clinical part of the course was very brief - less than one week, mostly only a few hours (n = 10). In total, 50 universities (19%) thus had no or a very brief GP/FM curriculum. These were mainly located in the Eastern or Southern European regions.

**Conclusion:**

It is still possible to graduate from European medical universities without having been exposed to a GP/FM curriculum. The European Academy of Teachers in General Practice (EURACT) will launch efforts to change this situation.

## Background

General practice/family medicine (GP/FM) is the provision of first contact, person focused, ongoing care over time that meets the health-related needs of people, referring only patients with uncommon or serious conditions, and coordinating care when people receive services at other levels of the healthcare system [1]. Primary health care means GP/FM applied on a population level, and as a population strategy this requires the commitment of governments to develop and sustain such services. It is increasingly becoming evident that a strong primary health care system is more likely to provide better population health, more equity in health throughout the population, and better use of economic resources, compared to systems that are oriented towards specialty care [2-4]. The World Health Organization (WHO) identified primary health care as central to the achievement of the goal “Health for All” already in 1978 [5], and thirty years later encouraged all countries to orient their health care systems towards a strengthened primary care [3].

To develop and maintain a strong and sustainable primary health care requires that a substantial part of graduating doctors go into primary care [3]. This in turn requires that GP/FM strongly influences the curricula in medical schools, although institutional, legislative and market factors also play important roles [6-8]. Specialty selection by medical students determines the future composition of the physician workforce. Among multiple reasons influencing a career choice either towards or away from primary care, medical school curricula may affect students in their perceptions of the role of primary care physicians. Since students are greatly influenced by the cultures of the institutions in which they are trained, the negative attitude of a university towards FM/GP may negatively affect the number of students going into this specialty [6]. Selection of career specialties begins in earnest during the clinical rotations with exposure to the clinical and intellectual environments of various specialties. A recent study from Israel found that as many as 62% of last year medical students considered choosing one specific specialty, while the rest considered two or more [9].

Still, however, undergraduate medical education seems to be out of synchrony with accelerating development and training in GP/FM [8]. In a former paper, our group developed a “minimal core curriculum” for undergraduate GP/FM, meant as an aid for medical schools introducing the topic for the first time, usually starting with a very brief course [10]. Working in a GP/FM setting requires problem solving skills, that differ highly from the disease-centered linear thinking inside university hospitals and which dominates the curricula in medical schools. In primary care the focus is upon the whole person – body and mind – in his/her context, and over long periods of time. Complex and poorly understood health problems, as well as clusters of diseases have to be handled, and the doctor-patient relationship is an important working tool. No student should graduate from medical school without substantial understanding of these matters [8,10]. In our opinion this requires – in addition to a theoretical GP/FM curriculum - a substantial component of “master-apprentice” learning in a primary care clinic.

In the last decades many European countries have undergone dramatic changes, including democratization, economic liberalization and a redefinition of the role of the state. Health care and social services systems have been reformed, and new challenges have had to be faced [4,11]. In the present paper, we aim at assessing and describing the extent of GP/FM teaching in medical schools throughout Europe. Is there a GP/FM curriculum? And to which degree are the students able to participate in clinical work in a GP’s office? Is it still possible to graduate as a doctor from a European university having learned nothing at all about GP/FM? We have not been able to find previous surveys on this matter.

## Methods

The authors of this paper are national representatives in the Council of European Academy of Teachers in General Practice and Family Medicine (EURACT) [12], and are members of EURACT’s Basic Medical Education (BME) Committee. Following a brain-storm within the BME Committee items for a questionnaire were identified. To achieve the best possible response rate the questionnaire was made as brief and simple as possible (Table 1) and was accompanied by the following text: “The European Academy of Teachers in General Practice/Family Medicine (EURACT) is running a mapping of the presence of undergraduate FM/GP rotations/clerkships in all European Medical Schools. You are kindly requested to answer the attached short questionnaire about your own medical school”. The questionnaire was sent by e-mail to GP/FM professors at each medical school in the countries of interest (where there were no GP/FM professors, the dean or another relevant professor was contacted). The authors divided the countries between them, and in several countries the national EURACT representative – not members of the BME-committee – aided in distributing the questionnaires and collecting the answers. Generally we accepted the data as they arrived from the respondents. The data collection took place in 2011 and until the end of 2012.

**Table 1:** **Questionnaire presented by The European Academy of Teachers in General Practice/Family Medicine (EURACT**)

***Question***	***Answer***
Name of medical school	
City	
How many years is your medical program?	
Does the school have a GP/FM curriculum?	
If so, does it have a clinical component (student sits in with GP)?	
What is the duration of this rotation in weeks?	
During which year is the rotation presented?	
Do you have such rotations in more than one year?	

As the study did not collect data on human subjects, no approval from an ethics committee was needed.

## Results

We were able to obtain information from 259 European medical schools in 39 countries (Tables 2, 3, 4 and 5). According to the “Avicenna Database” run by the University of Copenhagen in collaboration with World Federation for Medical Education (WFME), there are about 400 medical universities in these 39 European countries (http://avicenna.ku.dk/database/medicine). Response rate was thus 64%.

**Table 2:** Western Europe – state of GP/FM curriculum in medical schools

***Country (n = 5)***	***GP curriculum***	***Clinical component***	
***Medical school (n = 53)***	***Yes/no***	***Weeks n***	***Which year***
*Austria*			
Medical University Graz	Yes	5	6^th^
Medical University Innsbruck	Yes	4	6^th^
Medical University Vienna	Yes	3	5^th^ + 6^th^
Medical University Salzburg	Yes	4	5^th^
*Belgium*			
K.U. Leuven	Yes	10	2^nd^ + 6^th^
Univ Gent	Yes	10	2^nd^,3^rd^, 6^th^
VUB Brussels	Yes	12	2^nd^,5^th^, 6^th^
UA Antwerp	Yes	8	3^rd^, 5^th,^ 6^th^
UCL Brussels	Yes	2	3^rd^
ULB Brussels	Yes	5	3^rd^ + 6^th^
ULG Liege	Yes	5	2^nd^ + 6^th^
*Netherlands*			
Maastricht	Yes	10	5^th^
*Germany*			
Univ. Aachen	Yes	1	5^th^
Berlin	Yes	1	5^th^
Bochum	Yes	2	4^th^
Bonn	Yes	1	5^th^
Dresden	Yes	1	5^th^
Düsseldorf	Yes	2	5^th^
Erlangen	Yes	1	5^th^
Essen	Yes	2	4^th^
Frankfurt	Yes	2	5^th^
Freiburg	Yes	2.5	5^th^
Giessen	Yes	2	4^th^
Göttingen	Yes	2	5^th^
Greifswald	Yes	1	4^th^
Halle	Yes	2	5^th^
Hamburg	Yes	1	4^th^
Hannover	Yes	2	5^th^
Heidelberg	Yes	2	1^st^ or 2_nd_ + 4_th_
Homburg	Yes	1	No information
Jena	Yes	2	4^th^
Kiel	Yes	2	4^th^
Köln	Yes	2	4^th^ or 5^th^
Leipzig	Yes	2	4^th^
Lübeck	Yes	1	No information
Magdeburg	Yes	2-3	4^th^ + 5^th^
Mainz	Yes	1	5^th^
Mannheim	Yes	No information	
Marburg	Yes	2	5^th^
München (LMU)	Yes	1	4^th^
München (TU)	Yes	2	4^th^ + 5^th^
Münster	Yes	2	4^th^
Regensburg	Yes	?	
Rostock	Yes	1	5^th^
Tübingen	Yes	2	5^th^
Ulm	Yes	2	4^th^ + 5^th^
Witten/Herdecke	Yes	8-10	1^st^ to 5^th^
Würzburg	Yes	5	5^th^
*Switzerland*			
Univ of Basel	Yes	4	4^th^
Univ of Bern	Yes	6	1^st^, 2^nd^, 3^rd^, 4^th^, 6^th^
Univ of Geneva	Yes	3	2^nd^, 4^th^, 5^th^
Univ of Lausanne	Yes	5	3^rd^, 4^th^, 5^th^, 6^th^
Univ of Zürich	Yes	2	3^rd^, 4^th^, 5^th^

**Table 3:** Eastern Europe - state of GP/FM curriculum in medical schools

***Country (n = 10)***	***GP curriculum***	***Clinical component***	
***Medical school (n = 50)***	***Yes/No***	***Weeks***	***Which year***
*Belarus*			
Minsk State Medical University^1^	No		
Vitebsk State Medical University^1^	No		
Gomel State Medical University^1^	No		
GrodNo State Medical University^1^	No		
*Bulgaria*			
Medical University Plovdiv^1^	No		
Medical University Sofia	Yes	2	6^th^
Medical University Varna^1^	No		
Medical University Pleven^1^	No		
Medical faculty Stara Zagora^1^	No		
*Check Republic*			
Charles Univ in Prague, first fac of med	Yes	3	4^th^, 6^th^
Charles Univ in Prague, second fac of med	Yes	1	6^th^
Charles Univ in Prague, third fac of med	Yes	1	6^th^
Charles Univ, fac of med Hradec Kralove^2^	Yes	3 h	6^th^
Fac of med in Pilsen, Masaryk University	Yes	1	5^th^ + 6^th^
Fac of med , Palacky Univ Olomouc	Yes	1	6^th^
Univ Ostrava, fac med^1^	No		
*Georgia*			
Akaki Tsereteli State Univ, Caucasus	Yes	1	6^th^
International Univ Tbilisi^1^	No		
David Agmashenelebi Univ of Georgia	Yes	2	5^th^
David Tvildiani Med Univ	Yes	2	6^th^
Iv. Javakhishvili Tbilisi State Univ	Yes	2	5^th^
Petre Shotadze Tbilisi Med Acad	Yes	2	6^th^
Shota Rustaveli State Univ^1^	No		
Tbilisi Med EduUniv Hippocrates	Yes	1.5	6^th^
Tbilisi State Med Univ	Yes	2	1^st^, 2^nd^
*Hungary*			
Semmelweis Univ Budapest	Yes	1	6^th^
Univ of Szeged	Yes	1	6^th^
Univ of Pecs	Yes	1.5	6^th^
*Moldova*			
Univ Nicolae Testemitanu Chisinau	Yes	3	5^th^
*Poland*			
Med Univ of Bialystok^2^	Yes	2 h	6^th^
Wroclaw Med Univ^2^	Yes	5 h	6^th^
Med Univ of Gdansk	Yes	2.5	6^th^
Med Univ of Silesia, School of Med in Katowice	Yes	6	6^th^
Med Univ of Lodz	Yes	4	5^th^
Med Univ of Lublin	Yes	2.5	6^th^
Poznan Univ of Med Sciences^2^	Yes	<1	6^th^
Pomorski Univ of Med Stettin	Yes	2.5	6^th^
Med Univ of Warsaw	Yes	2.5	6^th^
Ludwig Rydygier CollMed Bydgoszcz	Yes	2	6^th^
*Romania*			
Gr. T. Popa, Univ of Med, Lasi	Yes	6	6^th^
Fac de Med Victor Papilian, Sibiu^1^	No		
Univ Transilvaia, Brasov^2^	Yes	No	
Univ Med Pharm, Victor Babes, Timisoara^2^	Yes	No	
Univ Med Pharm Iuliu Hatieganu, Cluj-Napoca	Yes	2.5	6^th^
*Russia*			
KrasNoyarsk^2^	Yes	30 h	6^th^
State Med Univ Vladivostok	Yes	1.5	6^th^
Amurskaya State Med Acad Blagoveshensk	Yes	1	6^th^
State Med Univ Kursk^2^	Yes	6 h	6^th^
State Med Univ Petrozavodsk^2^	Yes	6 h	6^th^
Pavlov’s St.Petersburg State Med Univ	Yes	4	5^th^, 6^th^
State North-West Med Univ St. Petersburg	Yes	2	6^th^
*Slovakia*			
Pavol Josef Safarik Univ Kosice	Yes	1	4^th^
Jessenius Fac Med Martin	Yes	2	5^th^
Comenius Univ Bratislava	No		

^1^No GP/FM curriculum (n = 13).

^2^No or < 1 week clinical component (n = 9).

**Table 4:** Northern Europe – state of GP/FM curriculum in medical schools

***Country (n = 9)***	***GP curriculum***	***Clinical component***	
***Medical school (n = 45)***	***Yes/no***	***Weeks n***	***Which year?***
*Denmark*			
Aarhus Univ	Yes	3	6^th^
Univ of south Denmark Odense	Yes	6	6^th^
Aalborg Univ	Yes	2	4^th^ or 5^th^
Copenhagen Univ	Yes	No information	
*Estonia*			
Univ of Tartu	Yes	6	1^st^, 6^th^
*Finland*			
Univ of Helsinki	Yes	4.5	1^st^, 2^nd^, 4^th^,5^th^
Univ of Kuopio	Yes	9	1^st^, 2^nd^, 3^rd^, 5^th^, 6^th^
Univ of Oulu	Yes	4	1^st^, 2^nd^, 5^th^, 6^th^
Univ of Tampere	Yes	5	3^rd^, 4^th^, 5^th^, 6^th^
Univ of Turku	Yes	4.5	1^st^, 3^rd^, 5^th^
*Iceland*			
Med School of Iceland Reykjavik	Yes	4	2^nd^, 6^th^
*Ireland*			
Univ of Limerick	Yes	?	1^st^, 4^th^
Royal College of Surgeons Med School	Yes	3	1^st^, 4^th^
Queens Univ Belfast	Yes	4	4^th^
Trinity College, Dublin	Yes	4	4^th^
NUI Galway	Yes	6	1^st^,2^nd^, 4^th^
Univ College Cork	Yes	7	3^rd^, 5^th^
*Latvia*			
Riga Stradins Univ	Yes	4	6^th^
Univ of Latvia	Yes	4	6^th^
*Norway*			
Univ of Bergen	Yes	4	6^th^
Univ of Oslo	Yes	7	1^st^, 2^nd^, 5^th^
Univ of Tromsø	Yes	8	1^st^, 5^th^
Norw Univ of Science and Technol, Trondheim	Yes	7	1^st^, 2^nd^, 6^th^
*Sweden*			
Sahlgrenska Acedemy Gothenburg	Yes	6	1^st^, 2^nd^, 3^rd^, 5^th^
Linköping Univ	Yes	12	years 1-6
Örebro univ	Yes	12	years 1-6
Umeå Univ	Yes	6	3^rd^, 4^th^, 6^th^
Karolinska Inst Stockholm	Yes	13	years 1-6
*United Kingdom*			
Keele Univ	Yes	23	3^rd^, 4^th^, 5^th^
NewcastleMed School	Yes	8	years 1-5
Barts and The London	Yes	5+	years 1-5
Edinburgh	Yes	7	4^th^, 5^th^
Brighton and Sussex Med School	Yes	4++	1^st^, 2^nd^, 4^th^, 5^th^
Cambridge	Yes	12+	4^th^, 5^th^, 6^th^
Lancaster Med School	Yes	15	years 2-5
Leicester	Yes	7	4^th^
Nottingham	Yes	4+	5^th^
King’s College, London	Yes	10	years 1-5
Dundee Med School	Yes	12	4^th^, 5^th^
Bristol	Yes	7-8	years 1-5
Univ of East Anglia	Yes	19	years 1-5
St. George’s Univ of London	Yes	9	3^rd^, 5^th^
Birmingham	Yes	9	years 1-5
Glasgow	Yes	20	3^rd^, 4^th^, 5^th^
Warwick Med School	Yes	8	2^nd^, 3^rd^

**Table 5:** Southern Europe – state of GP/FM curriculum in medical schools

***Country (n = 15)***	***GP curriculum***	***Clinical component***	
***Medical school (n = 107)***	***Yes/No***	***Weeks n***	***Which year?***
*Albania*			
Univ of Tirana^1^	No		
*Bosnia-Herzegovina*			
Med fak Banja Luka	Yes	3	5^th^
Med fak Tuzla	Yes	No information	
*Croatia*			
Rijeka	Yes	2	6^th^
Zagreb	Yes	2	6^th^
Osijek	Yes	2	6^th^
Split	Yes	2	6^th^
*Cyprus*			
Univ of Nicosia^1^ (only first two years of med school)	No		
*Greece*			
Athens^1^	No	4	6^th^
Aristotle Univ of Thessaloniki	Yes		1^st^
Patras^1^	No	4	6^th^
Heraklion, Crete	Yes	2	
Ioannina	Yes		
Alexandroupoli^1^	No		
Larissa^1^	No		
*Italy*			
Univ of L’Aquila	Yes	1	1^st^, 6^th^
Fac La Sapienza	Yes	4	6^th^
Fac di Med et Psicol Roma^2^	Yes	30 h	5^th^
Campus Biomedico Roma	Yes	1	5^th^
Univ of Udine	Yes	2	6^th^
Univ of Trieste^1^	No		
Central Milan	Yes	1	5^th^
S.Paolo Milan	Yes	1	5^th^
Vialba Milan	Yes	1	5^th^
S.Donato Milan	Yes	1	5^th^
Univ of GeNoa	Yes	2	6^th^
Univ of Bari et Foggia	Yes	2	6^th^
*Macedonia*			
Univ SS Cyril & Methodius Skopje	Yes	1	6^th^
State Univ Tetovo^2^	Yes	15 h	6^th^
Univ Goce Delcev Stip^2^	Yes	15 h	6^th^
*Malta*			
Univ of Malta	Yes	4	4^th^
*Montenegro*			
Podgorica	Yes	1	4^th^, 5^th^
*Portugal*			
Univ da Coimbra	Yes	10	6^th^
Univ da Lisboa	Yes	10	1^sr^, 2^nd^, 6^th^
Univ da Porto	Yes	4	6^th^
Univ da Beira Interior	Yes	4	1^sr^, 2^nd^, 4^th^, 5^th^, 6^th^
Univ da Minho	Yes	16	5^th^, 6^th^
Univ da Algarve	Yes	16	1^st^, 2^nd^, 3^rd^
*Serbia*			
Med Fak Nis	Yes	1	5^th^
Med Fak Belgrade	Yes	3	6^th^
Med Fak Kragujevac^1^	No		
Med Fak Novi Sad^1^	No		
Relocated Med Fak from Pristina^1^	No		
*Slovenia*			
Ljubljana	Yes	7	6^th^
*Spain*			
Cadiz	Yes	4	
Cordoba^2^	Yes	No	
Granada^2^	Yes	No	6^th^
Sevilla	Yes	3	
Zaragoza	Yes	4	6^th^
Asturias^2^	Yes	No	
La Laguna	Yes	6	5^th^, 6^th^
Las Palmas	Yes	8	6^th^
Cantabria	Yes	?	
Salamanca	Yes	3	6^th^
Valladolid	Yes	4	3^rd^, 6^th^
Albacete	Yes	6	6^th^
UAB-Univ AutoNoma Barcelona	Yes	11	1^st^
Univ Barcelona	Yes	4	5^th^
Girona	Yes	2	3^rd^, 4^th^, 5^th^ 6^th^
Lleida	Yes	6	6^th^
Rovira I Virgili	Yes	2	6^th^
Extremadura	Yes	8	
Santiago	Yes	4	3^rd^
Univ AutoNoma de Madrid	Yes	4	6^th^
Complutense de Madrid	Yes	4	5^th^ or 6^th^
Europ Univ Madrid	Yes	No information	
Alfonso	Yes	2	
Murcia	Yes	4	6^th^
Navarra	Yes	4	6^th^
Valencia	Yes	1	3^rd^
Catholic Univ Valencia	Yes	4	3^rd^, 4^th^
Miguel Hernandez, San Juan	Yes	6	
Pais Vasco	Yes	2	6^th^
*Turkey*			
Acibadem Istanbul	Yes	13	1^st^, 2^nd^, 3^rd^, 6^th^
Cukorova Adana	Yes	3	6^th^
Kocatepe Afyon^1^	No		
Ondokuzmayis Samsun	Yes	4	6^th^
Osmangazi Eskisehir^1^	No		
Selcuk Meram Konya^1^	No		
Sutcu Imam Kahramanmaras	Yes	4	6^th^
Trakya Edirne	Yes	2	4^th^
Uludag Bursa	Yes	1	6^th^
Tayfur Ata SokmenHatay	Yes	4	6^th^
Bozok Yozgat^1^	No		
Gulhane Askeri Tip Akademisi	No		
Ankara^1^			
Ankara Univ	Yes	1	5^th^
INonu Malatya	Yes	4	6^th^
Marmara	Yes	8	3^rd^,5^th^, 6^th^
Pamukkale Denizli^1^	No		
Mersin^1^	No		
Dokuz Eylül Izmir^1^	No		
Onsekiz Mart Canakkale	Yes	8 (elective)	5^th^, 6^th^
Yeditepe Istanbul	Yes	6	6^th^
Adnan Menderes Aydin	Yes	5	5^th^, 6^th^
Akdeniz Antalya	Yes	5	3^rd^, 6^th^
Baskent Ankara	Yes	2	4^th^
Izzet Baysal Abant^1^	No		
Karadeniz Techn Univ Trabzon^1^	No		
Trabzon^1^			
Celal Bayar Manisa^1^	No		
Yildirim Beyazit Ankara	Yes	4	6^th^
Capa Istanbul^1^	No		
Gazi Osman Pasa Tokat	Yes	4	5^th^
*Israel*			
Ben –Gurion Univ Beer-Sheva	Yes	6	6^th^
Hebrew Univ Jerusalem	Yes	2	6^th^
Tel-Aviv Univ (6 y med school)	Yes	4	6^th^
Tel-Aviv Univ (4 y med school)	Yes	3	4^th^
Technion Haifa	Yes	6	6^th^

^1^No GP/FM curriculum (n = 22).

^2^No or < 1 week clinical component (n = 6).

Out of the 259 respondent universities, 35 (13.5%) reported to have no GP/FM curriculum (Table 6). These 35 medical faculties were located in eleven different European countries. In addition, 15 of the medical schools where a GP/FM curriculum did exist, reported that this curriculum did not include any clinical component (n = 5), or that the clinical part of the course was very brief - less than one week, mostly only a few hours (n = 10, Table 6). In total, 50 universities (19%) thus had no or a very brief GP/FM curriculum.

**Table 6:** Medical universities without GP/FM curriculum, or with clinical GP/FM teaching lacking or shorter than one week

***Country***	***Medical schools without GP/FM curriculum (n = 35)***
*Belarus*	Minsk State Medical University
	Vitebsk State Medical University
	Gomel State Medical University
	Grodno State Medical University
*Bulgaria*	Medical University Plovdiv
	Medical University Varna
	Medical University Pleven
	Medical faculty Stara Zagora
*Chech Republic*	Univ. Ostrava, fac med
*Georgia*	International Univ Tbilisi
	Shota Rustaveli State Univ
*Romania*	Fac de Med Victor Papilian, Sibiu
*Slovakia*	Comenius Univ Bratislava
*Albania*	University of Tirana
*Cyprus*	University of Nicosia (only first two years of med school)
*Greece*	Athens
	Patras
	Alexandroupoli
	Larissa
*Italy*	University of Trieste
*Serbia*	Med Fak Kragujevac
	Med Fak Novi Sad
	Relocated Med Fak from Pristina
*Turkey*	Kocatepe Afyon
	Osmangazi Eskisehir
	Selcuk Meram Konya
	Bozok Yozgat
	Gulhane Askeri Tip Akademisi Ankara
	Pamukkale Denizli
	Mersin
	Dokuz Eylül Izmir
	Izzet Baysal Abant
	Karadeniz Techn Univ Trabzon
	Celal Bayar Manisa
	Capa Istanbul
	***Medical schools with no or <1 week clinical teaching (n = 15)***
*Chech Republic*	Charles Univ, fac of med Hradec Kralove
*Poland*	Med Univ of Bialystok
	Wroclaw Med Univ
	Poznan Univ of Med Sciences
*Romania*	Univ Transilvaia, Brasov
	Univ Med Pharm, Victor Babes, Timisoara
*Russia*	Krasnoyarsk
	State Med Univ Kursk
	State Med Univ Petrozavodsk
*Italy*	Fac di Med et Psicol Roma
*Macedonia*	State Univ Tetovo
	Univ Goce Delcev Stip
*Spain*	Cordoba
	Granada
	Asturias

Tables 2, 3, 4 and 5 show details of the GP/FM curriculum in the medical schools in the four European regions (according to the United Nations’ Geo scheme and also including Israel). Roughly, the comprehensiveness of the GP/FM curriculum varies between the regions, as all faculties without any such curriculum are located either in Eastern or in Southern Europe, as are also the majority of schools without or with a very short clinical GP/FM component. Only few medical schools in Eastern Europe have a rotation period longer than two weeks, while the majority of schools in Northern Europe have at least five weeks, and several up to 12–13 weeks.

There are substantial variations in length of the clinical component within countries and even inside the same city: for example the time spent in a GP’s office is two weeks in one Brussels medical school and 12 weeks in another.

## Discussion

One limitation of this study is that by labelling curricula as including or not including GP/FM, we assume the curricula to be mainly discipline based. We thus may have overlooked that a problem based or case based curriculum could include elements from GP/FM without having a proper GP/FM section. We also are aware of the fact that the mere existence of a GP/FM curriculum is not synonymous with high quality teaching. A further limitation is that we were not able to obtain data at all from some countries: Ukraine, Lithuania and France. These countries are “white spots on our map”, although we have got information from a key informant in France that all Universities have incorporated FM/GP in their BME curriculum and that all of them have a clinical component (2–6 weeks). This information is in line with the rest of Western European universities, as all of them have GP/FM training with a clinical component. Also, not all universities have responded from each country. For example, we got data only from seven universities in Russia, while the number of medical schools in this country is more than 60. Similarly, for Turkey we have data from 29 universities out of more than 50. Therefore, we cannot provide statistically valid information on the situation in Europe as such. On the other hand, the information that we do have from 259 European medical schools, in itself brings new insights, although curricula are in constant evolution and data captured at a certain point of time will not reflect such a dynamic situation.

In 2010 an independent international commission published a report on the need of transforming medical education in the future [13]. The commission states that professional education has not kept pace with the needs of patients and populations, and that fragmented and outdated curricula produce ill-equipped graduates. Reforms are therefore needed, and a list of ten proposed reforms is given. Point number eight states the need for medical schools to achieve: “Expansion from academic centers to academic systems, extending…….into primary care settings and communities…” [13]. It is thus positive that the majority of medical schools throughout the European regions do have a substantial GP/FM curriculum – 209 out of the 259 faculties assessed. Even so, there is ample room and need for improvements, as 35 schools have no GP/FM teaching whatsoever and clinical teaching is absent or very brief in several others.

Most former communist countries now let GP/FM play a key role in their health care system. A GP/FM curriculum is also increasingly being introduced into medical training at undergraduate and postgraduate level, and GP/FM is developed as an academic discipline [11,14]. Our study revealed that this task can not yet be seen as completed. It is especially worrying if it is possible both to graduate without any GP/FM competence and subsequetly set up a practice in a country without a mandatory vocational training program.

European primary care is currently facing high expectactations, regarding its promises to improve population health, control costs, and attribute to less socioeconomic inequality of care [1-4]. But: Do strong primary care systems indeed perform better? And what determines how strong primary care is? [15]. These important questions have recently been addressed by means of a EU-funded project: the Primary Health Care Activity Monitor for Europe (PHAMEU) [16]. Based on information from 31 European countries the study was able to show that strong primary care indeed was associated with better population health outcomes, lower rates of potentially avoidable hospitalization, lower socioeconomic inequality in self-rated health, a reduced growth of health care spending, but also with higher levels of total health care costs [17]. In total, this should support the efforts of policy makers to prioritize primary care. Development of primary care workforce is part of such efforts [18], and developing a comprehensive GP/FM undergraduate curriculum comprising a clinical rotation is a necessity in this process [6-8].

In our study most clinical GP/FM rotations were placed in years four, five or six, but as the length of the clinical teaching period increased, it is common to spread it over several semesters. For example the 12–13 weeks of rotations at three Swedish universities involve the years one to six. This probably has a positive influence on recruitment to GP/FM, as students are exposed to role models throughout their entire education [6-8]. In our opinion GP/FM should be positioned as one of the main clinical topics in every European medical school, and teaching in a one-to-one situation in a GP’s office should be offered for at least four weeks, preferably longer.

## Conclusion

Although the majority of the assessed universities reported to have a theoretical GP/FM curriculum as well as a clinical rotation, it is still possible to graduate from some European medical schools without having learned about clinical work in a primary care setting. The European Academy of Teachers in General Practice (EURACT) will lance efforts to change this situation. Special efforts should be made in Eastern and Southern Europe, where a FM/GP curriculum does not exist in several universities, and where the clinical GP/FM component is generally short.

## Competing interests

The authors declare that they have no competing interests.

## Authors’ contributions

The group of authors planned the study together, and all contributed to data collection. MB drafted the paper, with contribution from the group. All authors have given final approval of the version to be published.

## Pre-publication history

The pre-publication history for this paper can be accessed here:

http://www.biomedcentral.com/1472-6920/13/157/prepub
